# A Reliable Auto-Robust Analysis of Blood Smear Images for Classification of Microcytic Hypochromic Anemia Using Gray Level Matrices and Gabor Feature Bank

**DOI:** 10.3390/e22091040

**Published:** 2020-09-17

**Authors:** Bakht Azam, Sami Ur Rahman, Muhammad Irfan, Muhammad Awais, Osama Mohammed Alshehri, Ahmed Saif, Mohammed Hassan Nahari, Mater H. Mahnashi

**Affiliations:** 1 Department of Computer Science and IT, University of Malakand, Chakdara 18801, Pakistan; bazam@uom.edu.pk; 2 College of Engineering, Electrical Engineering Department, Najran University, Najran 61441, Saudi Arabia; irfan16.uetian@gmail.com; 3 School of Computing and Communications, Lancaster University, Bailrigg, Lancaster LA1 4YW, UK; m.awais11@lancaster.ac.uk; 4 Department of Clinical Laboratory Sciences, College of Applied Medical Sciences, Najran University, Najran 61441, Saudi Arabia; Omalshehri@nu.edu.sa (O.M.A.); amsaif8080@gmail.com (A.S.); Mhnahari@nu.edu.sa (M.H.N.); 5 Department of Medicinal Chemistry, Pharmacy School, Najran University, Najran 61441, Saudi Arabia; matermaha@gmail.com

**Keywords:** erythrocytes, RBCs, segmentation, classification, anemia, reliable

## Abstract

Accurate blood smear quantification with various blood cell samples is of great clinical importance. The conventional manual process of blood smear quantification is quite time consuming and is prone to errors. Therefore, this paper presents automatic detection of the most frequently occurring condition in human blood—microcytic hyperchromic anemia—which is the cause of various life-threatening diseases. This task has been done with segmentation of blood contents, i.e., Red Blood Cells (RBCs), White Blood Cells (WBCs), and platelets, in the first step. Then, the most influential features like geometric shape descriptors, Gray Level Co-occurrence Matrix (GLCM), Gray Level Run Length Matrix (GLRLM), and Gabor features (mean squared energy and mean amplitude) are extracted from each of the RBCs. To discriminate the cells as hypochromic microcytes among other RBC classes, scanning is done at angles (0${}^{\circ }$, 45${}^{\circ }$, 90${}^{\circ }$, and 135${}^{\circ }$). To achieve high-level accuracy, Adaptive Synthetic (AdaSyn) sampling for imbalance learning is used to balance the datasets and locality sensitive discriminant analysis (LSDA) technique is used for feature reduction. Finally, upon using these features, classification of blood cells is done using the multilayer perceptual model and random forest learning algorithms. Performance in terms of accuracy was 96%, which is better than the performance of existing techniques. The final outcome of this work may be useful in the efforts to produce a cost-effective screening scheme that could make inexpensive screening for blood smear analysis available globally, thus providing early detection of these diseases.

## 1. Introduction

**Figure 1:**
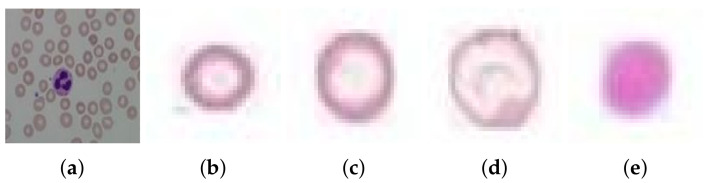
(**a**) A normal blood smear image and (**b**) microcytic hypochromic, (**c**) normocytic hypochromic, (**d**) macrocytic hypochromic, and (**e**) microcytic hyperchromic anemia.

Anemia is an abnormal condition in human blood in which the amount of red blood cells (and therefore their oxygen-carrying capacity) is inadequate to fulfill the physiologic needs of the body. The World Health Organization (WHO) estimates that 42% of children less than 5 years of age and 40% of pregnant women worldwide are anaemic [1,2]. According to the translated prevalence by [3], 496 million are nonpregnant women, 32 million are pregnant women, and 273 million are children with anemia in 2011. When a peripheral blood smear slide is viewed under a microscope, it is found to be composed of three main cell types, i.e., Red Blood Cells (RBCs), White Blood Cells (WBCs), and platelets. Normal RBCs are round and reddish in color, having a central pallor area in the center indicating hemoglobin level; see Figure 1. The diameter of normal RBCs ranges from about 6.2 to 8.2 micrometer (μm), and its thickness ranges from about 2 to 2.5 micrometers (μm) [4]. magenta The amount of hemoglobin indicates whether magenta a human is healthy or has a potential disease that affects RBC production or function. This indication is through several parameters of whole RBC (size, shape, and number) and the amount of hemoglobin it contains. The size of RBCs indicates three main defects in human blood: narmocytosis, microcytosis, and macrocytosis. Narmocytosis is an anemia of normocytes, where the size of RBCs is normal. Microcytosis occurs when the size of the RBC is less than that of the normal reference range and is expressed by a blood index called Mean Corpuscular Volume (MCV) of less than 80 femtoliters, whereas macrocytosis is when the size of the RBC is greater than the normal reference range and is expressed by an MCV greater than the normal range [5].

Patients of anemia may be categorized into mild, moderate, and severe according to WHO classification, i.e., mild anemia (hemoglobin 9.0–10.9 g/dL) moderate anemia (hemoglobin 7.0–8.9 g/dL), and severe anemia (hemoglobin <7.0 g/dL) [1]. The amount of hemoglobin on the other hand specifies two other abnormalities: hypochromic, if the mean concentration of hemoglobin in a single cell is lower than the normal reference range, i.e., 27 to 32 picogram (pg) (approximately) and is stated by Mean Corpuscular Hemoglobin Concentration (MCHC), and hyperchromic, if the range is above the said reference range [6].

The above discussed terms are parameters used in distinguishing the primary types of anemic conditions. When both cell size and hemoglobin defects are combined, e.g., if microcytic is taken from the first category and combined with hypochromic from the second category, then it will define the features of microcytic hypochromic anemia, whereas if macrocytic and hyperchromic are combined, it will give macrocytic hyperchromic anemia, etc. [7,8]. The diseases which are most frequently diagnosed in South Asian countries especially in Pakistan are iron deficiency anemia, sideroblastic anemia, and thalassemia, which are categorized and defined under microcytic hypochromic anemia [9]. These parameters may be obtained from peripheral blood smear slides prepared by hematologists in clinical labs of hospitals proceeded by grabbing digital images, which is an area of interest.

This work is organized as follows: related work is explained in Section 2, the preparation and visualization of the blood smear is explained in Section 3. To better understand the nature of improvement, we carried out a thorough study that builds up a methodology in which blood smear after following the necessary procedures is processed with image processing techniques for segmentation. The primary steps of image processing are image acquisition, preprocessing (conversion into color spaces, splitting channels, and enhancement techniques), binarization, segmentation, etc. [10]. Then, the classification step explained in Section 4 is conducted with Adaptive Synthetic (AdaSyn) sampling to balance the datasets and with Locality Sensitive Discriminant Analysis (LSDA) for the reduction of unnecessary features of all the contents visible in a blood smear. The rest of paper presents the results (Section 5) and finally the conclusion and discussion (Section 6).

## 2. Related Work

Various state-of-the-art image processing-based techniques for computer-aided disease detection are present excessively, some of which are the most relevant to our work presented here. Moallem et al. [11] used a three-step algorithm for segmentation of overlapped cells in blood smear images by extracting a binary mask in the first step, then an adaptive mean shift algorithm was used to centrally localize a cell, and finally the gradient vector flow algorithm was used to draw boundaries for separation of cells. In the given article, there is no shape analysis done, which fails to classify the significant classes of RBCs. Tomari et al. [12] used some geometric features for the classification of RBCs through Artificial Neural Network (ANN). Alomari et al. [13] worked on the automatic quantification of WBCs and RBCs by using an iterative structured circle detection algorithm, but in the given technique, it was found that it is restricted to circular cells. The technique suggested by Aggarwal et al. [14] is intensity-based Otsu thresholding where segmentation of RBCs infected with a parasite was done, but the segmentation by intensity may suffer extremely due to luminance variations and other photographic conditions. Tek et al. [15] suggested a technique for recognition and classification of malarial RBCs’ parasites and species in peripheral blood smears. Shape, color, and local granulometry features were extracted from the area of interest, and k Nearest Neighbor (kNN) classifier was applied to classify them from extracted features. Chen et al. [16] evaluated blood smear slides having hemolytic anemia by determining the chain codes for finding the edges of cells, separated the cells with the help of concavity measurement, and classified cells with a bank of classifiers. A work done by Xu et al. [17] for the detection of sickle cells is segmentation of red cells in the first step, then separation of overlapped cells using random walk algorithm in the next step, and classification through Deep Convolutional Network in the last step. Sharma et al. [18] used a median filter for image smoothing and watershed for overlapped cells separation. Then, they implemented a three-feature vector, circularity matric, aspect ratio, and radial signature, trained with KNN classifier for the recognition of three types of RBCs called sickle cells, elliptocytes, and dacrocytes. A work for the detection and classification of parasites in RBCs was done by Ahirwar et al. [19]. They generated geometric, color attributes and gray level texture feature sets and used artificial neural network for their classification. A fuzzy logic technique was used by Bhagavathi et al. [20] for segmentation of RBCs and WBCs. Morphological operations and hough transform method for circle detection were used by Chandrasiri et al. [21] for the detection and analysis of red blood cells, but the proposed system was unable to determine and analyze the extensive number of clumped or overlapped regions. The above presented approaches can do better in a situation where the population of clumped cells is low. In highly populated clumped cells, accuracy suffers. In our proposed approach, the issue is fixed to much extent by leveraging the concavities and texture-based features in each cell as explained in the following Materials and Methods section.

## 3. Materials and Methods

**Figure 2:**
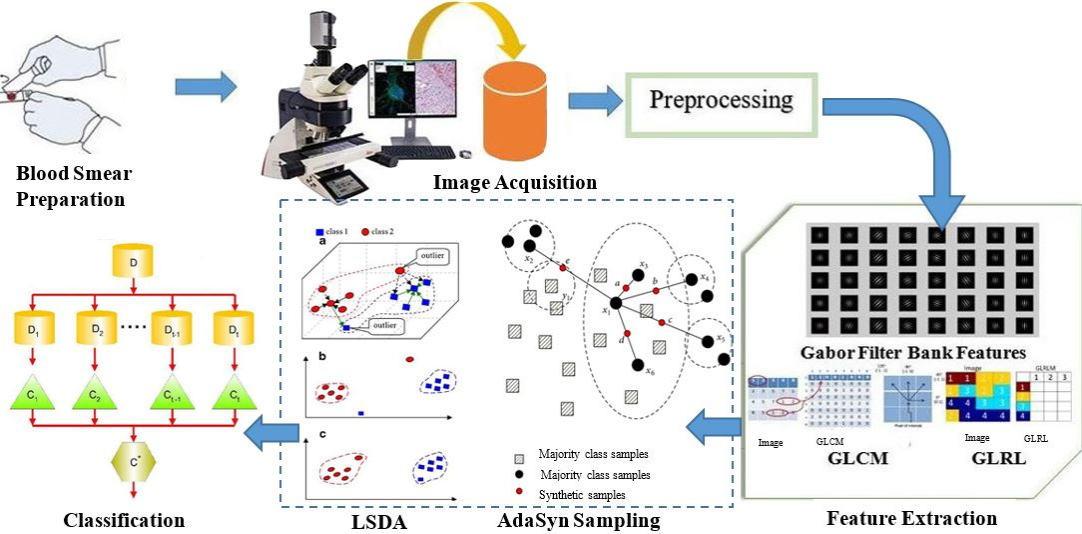
A step-by-step proposed plan of work.

Our proposed plan of work for the detection of hypochromic microcytic cells in sample blood smear images consisted of the following series of steps shown in Figure 2.

### 3.1. Blood Smear Slide Preparation

A consistent blood distribution and proper lucidity are required for reliable blood smear analysis. It can be done by starting with a drop of sample blood at one end of glass slide, which is smeared quickly and gently with a wedge technique to form a thin edge, where all cells are able to be analyzed separately, especially RBCs [22]. This whole process was done by an expert laboratory technician in the local hospital.

### 3.2. Image Acquisition

After the staining process was completed, the image acquisition step started by using a 400× field of a microscope with oil immersion, keeping the horizontal and vertical resolution at 180 dpi and the image dimension at 2592 × 1944 pixels. The images were captured and labelled properly.

### 3.3. Preprocessing

**Figure 3:**
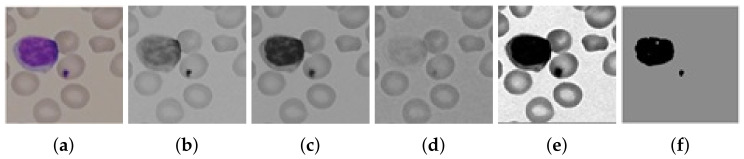
Output images after the preprocessing step: (**a**) original RGB image, (**b**) red channel, (**c**) green channel, (**d**) blue channel, (**e**) enhanced green channel, and (**f**) quantized image leaving behind RBCs.

A green channel of RGB blood smear was selected, enhanced, and smoothed using Balance Contrast Equalization Technique (BCET) and median filter. After getting a fine gray-scale image shown in Figure 3, it was quantized with a scale factor $F_{g}$, calculated from mean intensity value of g(x,y) calculated in Equations (1) and (2), where Q(x,y) is the resultant quantized image.

(1) $$F_{g}=\frac{1}{N}\sum_{1}^{N}g\left(x,y\right)$$

(2) $$Q\left(x,y\right)=F_{g}\nabla round\left(\frac{g(x,y)}{F_{g}}\right)$$

### 3.4. Segmentation

**Figure 4:**
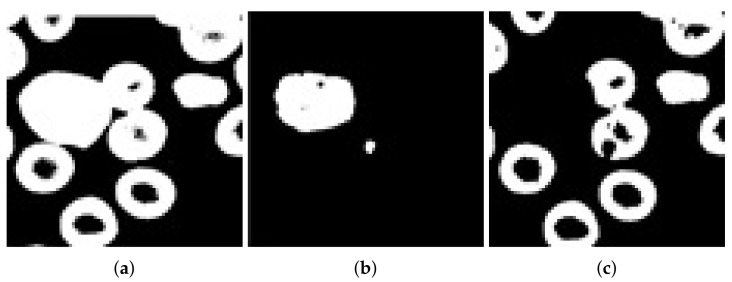
(**a**) Binarized original image of a sample blood smear, (**b**) binarized image of a sample blood smear quantized, and (**c**) exclusive OR of (**a**,**b**).

A global or automatic thresholding technique was used in this work for the segmentation of only RBCs (leaving behind WBCs and platelets). A two-step binarization technique was followed for getting noise-free binary images. In the first step, the quantized image was binarized containing white blood cells; in the second step, the whole original image was binarized. Finally, we used the XOR gate (Exclusive OR) operation to remove white blood cells. The output along with other processed images are shown in Figure 4.

### 3.5. Feature Extraction

A three-way feature extraction plan was implemented. The first was geometric morphological feature extraction, the second was intensity features and texture features using (GLCM and GLRLM), and the third was texture features using the Gabor Filter Bank.

#### 3.5.1. Geometric Morphology of Microcytic Hypochromic RBCs

Keeping in view the visual size and shape of hypochromic microcytes, the useful features were calculated as follows:

- Area: Area is an important geometrical feature for the detection of microcytes, being small in size compared to other blood cells.
- Circularity: A size-invariant shape descriptor given in Equation (3) which describes a shape to be circular, if the value is closer to 1 and noncircular if the value is closer to 0, where *A* is Area and *P* is parameter of a cell.
  (3) $$Circularity=\frac{4\pi A}{P^{2}}$$
- Rectangularity: It determines the degree of elongation with respect to a rectangle. Equation (4) shows its calculation, where $A_{s}$ is area of a shape and $A_{r}$ is the area of minimum bounding rectangle.
  (4) $$Rectangularity=\frac{A_{s}}{A_{r}}$$
- Concavity: This property is used to determine how much an object is concave; we applied it on the shapes for identification of the amount of central pallor area occupied in an RBC, given by Equation (5)
  (5) $$Concavity=\frac{A_{c}}{A_{H}}$$
- Convexity: A cell convexity can be determined by Equation (6), which identifies a shape through its boundary convexity.
  (6) $$Convexity=\frac{P_{C}H}{P_{C}}$$

#### 3.5.2. RBC Texture Feature Calculation

**Figure 5:**
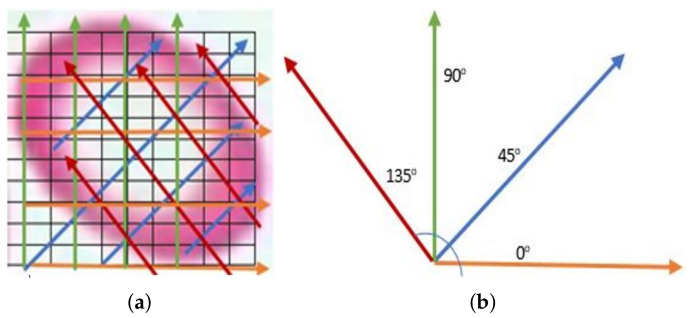
(**a**) Orientation of angles overlayed on a sample red blood cell (RBC) and (**b**) scanning through 0${}^{\circ }$, 45${}^{\circ }$, 90${}^{\circ }$, and 135${}^{\circ }$.

**Figure 6:**
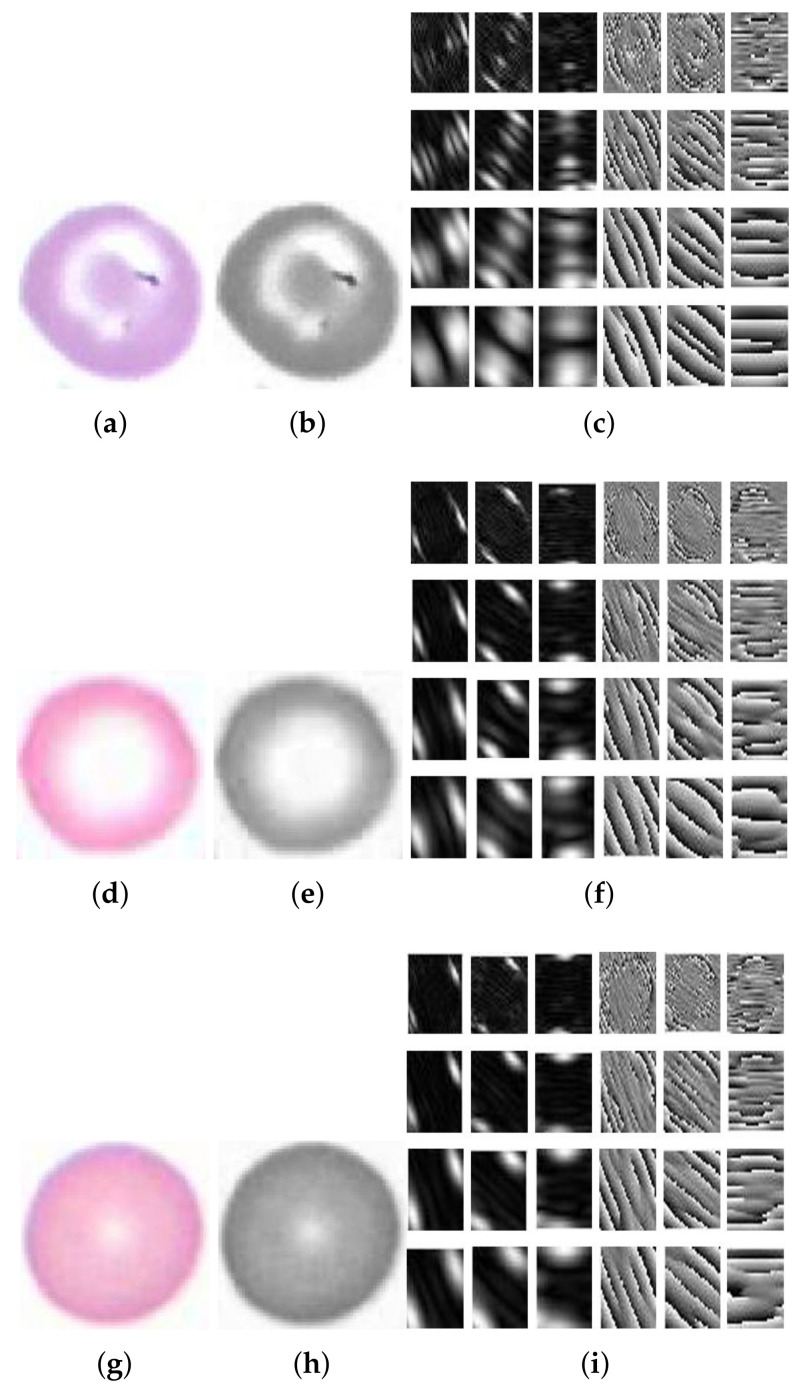
(**a**) Original image of hyperchromic macrocytic RBC, (**b**) gray level image of hyperchromic macrocytic RBC, (**c**) Gabor filter bank of hyperchromic macrocytic RBC, (**d**) original image of hypochromic microcytic RBC, (**e**) gray level image of hypochromic microcytic RBC, (**f**) Gabor filter bank features of hypochromic microcytic RBC, (**g**) original image of hyperchromic microcytic RBC, (**h**) gray level image of hyperchromic microcytic RBC, and (**i**) a hyperchromic microcytic RBC with its Gabor filter bank features.

Features other than geometry include texture information on RBCs. In this research work, we extracted RBG intensity-based features (mean and variances), Gray Level Co-occurrence Matrix (GLCM), Gray Level Run Length Matrix (GLRLM), and Gabor feature bank. This set of features associated with the dispersal of chromatin matter in the RBCs is helpful in the classification of hypochromic, hyperchromic, and normochromic cells given in Figure 1 above. The measure will indicate that either the RBC has a deep red color compared to the others (cells having more haemoglobin) or are less red, with more central pallor area and having a smaller haemoglobin ratio. The texture features calculated are presented below:

- RGB mean and variance of hypochromic microcytic RBCs: The mean values $\mu_{r}$, $\mu_{g}$, and $\mu_{b}$ of pixels of each RBC in R, G, and B, respectively, were calculated in Equation (7).
  (7) $$\begin{array}{c} \mu_{r}=\frac{1}{N}\sum_{1}^{N}r\left(x,y\right)\\ \mu_{g}=\frac{1}{N}\sum_{1}^{N}g\left(x,y\right)\\ \mu_{b}=\frac{1}{N}\sum_{1}^{N}b\left(x,y\right) \end{array}$$
  The variances ${\left(\sigma_{r}\right)}^{2}$, ${\left(\sigma_{g}\right)}^{2}$, and ${\left(\sigma_{b}\right)}^{2}$ in the channels R, G, and B, respectively, are calculated in Equation (8)).
  (8) $$\begin{array}{c} {\left(\sigma_{r}\right)}^{2}=\frac{\sum {\left(X-\mu_{r}\right)}^{2}}{N}\\ {\left(\sigma_{g}\right)}^{2}=\frac{\sum {\left(X-\mu_{g}\right)}^{2}}{N}\\ {\left(\sigma_{b}\right)}^{2}=\frac{\sum {\left(X-\mu_{b}\right)}^{2}}{N} \end{array}$$
- GLCM features of Hypochromic Microcytic RBCs: GLCM is the distribution of cooccurring pixel values defined over an N × N image P at a specific offset, or every P’s element determines the occurrences of a pixel with value of gray level, i, lifted by a certain distance to a pixel with value j. Our next six textural features are GLCM features. The mean of 6 GLCM features were determined for offset values conforming to 0${}^{\circ }$, 45${}^{\circ }$, 90${}^{\circ }$, and 135${}^{\circ }$ consuming 8 gray levels (see Figure 5).
  Maximum Probability: It measures the strongest response of the cooccurrence matrix. The range of values is [0, 1] as given in Equation (9), where $P_{ij}$ is the pixels of gray image.
  (9) $$P=max_{ij}\left(P_{ij}\right)$$
  Correlation: The degree of correlation of a pixel to its neighbor is determined by the correlation factor of the cooccurrence matrix, ranging from 1 to −1 given by Equation (10). This measure cannot be defined if any of the standard deviation σ is 0 for the two existing correlations, perfect positive and perfect negative correlation.
  (10) $$correlation=\sum_{i,j=1}^{K}\frac{\left(i-m_{r}\right)\left(j-m_{c}\right)p_{\left(ij\right)}}{\sigma_{r}\sigma_{c}}$$
  Pixels intensity contrast: It is a measure of intensity contrast between a pixel and its neighbor over the entire image (calculated in Equation (11)).
  (11) $$contrast=\sum_{i,j=1}^{k}{\left(i-j\right)}^{2}p_{ij}$$
  Energy: It is the measurement of uniformity in the intensities of an image (as given in Equation (12)). Its value is 1, if an image is constant and 0 if the intensities are variable.
  (12) $$Energy=\sum_{i,j=1}^{k}{\left(P_{\left(i,j\right)}\right)}^{2}$$
  Homogeneity: It measures the spatial closeness of the distribution of elements in the cooccurrence matrix to the diagonal given by (13). The values range is [0, 1], and the maximum value is attained when the matrix is a diagonal.
  (13) $$homogeneity=\sum_{i,j=1}^{k}\frac{P_{ij}}{1+|i-j|}$$
  Entropy: It measure the degree of variability of the elements of the cooccurrence matrix. Its value is 0 if all intensities of $P_{ij}$ are 0 and is maximum when all $P_{ij}$ are equal. It may be calculated by (14).
  (14) $$entropy=-\sum_{i,j=1}^{k}P_{ij}log_{2}P_{ij}$$
- Run length matrix features of each RBC: The other textural features are created on the gray-level run length matrix (calculated in Equations (15)–(24). The l∇K matrix *p*, where *l* is the number of gray levels and *k* is the maximum run length, is defined for a certain image as the total runs with pixels of gray level *i* and run length *j*. Likewise, as in the GLCM, the run length matrices were calculated using 8 gray-levels for 30${}^{\circ }$, 60${}^{\circ }$, 90${}^{\circ }$, and 135${}^{\circ }$.
  Short Run Emphasis (SRE):
  (15) $$SRE=\frac{1}{R}\sum_{i=1}^{k}\sum_{j=1}^{k}\frac{{}_{P_{ij}}}{j^{2}}$$
  Long Run Emphasis (LRE):
  (16) $$LRE=\frac{1}{R}\sum_{i=1}^{k}\sum_{j=1}^{k}\left(p_{i}j\right)j^{2}$$
  Gray-Level Nonuniformity (GLN):
  (17) $$GLN=\frac{1}{R}\sum_{i=1}^{k}{\left(\sum_{j=1}^{l}\left(p_{i}j\right)\right)}^{2}$$
  Run Length Nonuniformity (RLN):
  (18) $$RLN=\frac{1}{R}\sum_{j=1}^{l}{\left(\sum_{i=1}^{k}p_{ij}\right)}^{2}$$
  Low Gray-level Run Emphasis (LGRE):
  (19) $$LGRE=\frac{1}{R}\sum_{i=1}^{k}\sum_{j=1}^{l}\frac{pij}{i^{2}}$$
  High Gray-level Run Emphasis (HGRE):
  (20) $$HGRE=\frac{1}{R}\sum_{i=1}^{k}\sum_{j=1}^{l}\left(p_{ij}\right)i^{2}$$
  Short Run Low Gray-level Emphasis (SRLGE):
  (21) $$SRLGE=\frac{1}{R}\sum_{i=1}^{k}\sum_{j=1}^{l}\frac{p_{ij}}{i^{2}j^{2}}$$
  Short Run High Gray-level Emphasis (SRHGE):
  (22) $$SRHGE=\frac{1}{R}\sum_{i=1}^{k}\sum_{j=1}^{l}\frac{p_{\left(ij\right)}i^{2}}{j^{2}}$$
  Long Run Low Gray-level Emphasis (LRLGE):
  (23) $$LRLGE=\frac{1}{R}\sum_{i=1}^{k}\sum_{j=1}^{l}\frac{p_{\left(ij\right)j^{2}}}{i^{2}}$$
  Long Run High Gray-level Emphasis (LRHGE):
  (24) $$LRHGLE=\frac{1}{R}\sum_{i=1}^{k}\sum_{j=1}^{l}\left(p_{ij}\right)i^{2}j^{2}$$
- Gabor Feature Extraction: like a human visual processing system, the Gabor filter extracts features at different amplitudes and orientation.
  (25) $$\begin{array}{c} \psi \left(x,y\right)=\frac{f^{2}}{\pi \gamma \eta }e^{-}\left(\frac{f^{2}}{\gamma^{2}}x^{{}^{\prime }2}\right)+\left(\frac{f^{2}}{\eta^{2}}y^{{}^{\prime }2}\right)e^{j2\pi fx^{\prime }}\\ x^{\prime }=x\cos\theta +y\sin\theta \\ y^{\prime }=x\sin\theta +y\cos\theta \end{array}$$
  The Gabor filter is the product of a 2D Fourier basis function and origin-centred Gaussian given in Equation (25), where f is the central frequency of the filter, γ and η are the sharpness or bandwidth measure along the minor and major axes of Gaussian respectively, θ is the angle of rotation, and (η/γ) is the aspect ratio. The analytical form of this function in frequency domain is given in Equation (26) as follow:
  (26) $$\begin{array}{c} \psi \left(u,v\right)=e^{-\frac{\pi^{2}}{f^{2}}\left(\gamma^{2}{\left(u^{\prime }-f\right)}^{2}+\eta^{2}v^{{}^{\prime }2}\right)}\\ u^{\prime }=u\cos\theta +v\sin\theta \\ v^{\prime }=u\sin\theta +v\cos\theta \end{array}$$
  In the frequency domain given by Equation (27), the function is a single real-valued Gaussian centered at f. A simplified version of a general 2D Gabor filter function in Equations (25) and (26) was formulated by [23], which implements a set of self-similar filters, i.e., Gabor wavelets (rotated and scaled forms of each other, irrespective of the frequency *f* and orientation θ.
  Gabor bank or Gabor features were created from responses of Gabor filters in Equations (25) and (26) by using multiple filters on several frequencies $f_{m}$ and orientations $\theta_{n}$. Frequency in this case corresponds to scale information and is thus drawn from [23]
  (27) $$f_{m}=k^{-m}f_{m}ax,m=\left\{0,\ldots ,M-1\right\}$$
  where fm is the mth frequency, $f\theta =f_{m}ax$ is the highest frequency desired, and k>1 is the frequency scaling factor. The filter orientations are drawn from [24]. Gabor features were calculated at 4 wavelengths (3, 6, 9, and 12) and 3 orientations θ (30${}^{\circ }$, 60${}^{\circ }$, and 90${}^{\circ }$); see Figure 6a–c. Then, each filter was convolved with the real image, and the response image of the same image was produced; here, each image gave us a feature vector. Each feature vector consisted of mean amplitude and mean squared energy. Finally, two matrices were obtained, that were of [1 × 12] each. The matrices were appended to each other, and a [1 × 24] matrix was produced for one image having a [n × 24] vector for n images for supplementary training purpose in the preceding step of classification (as shown in Figure 6).

### 3.6. ADASYN Sampling

A significant aspect in classification and learning is to show a reasonable dataset to guarantee that no inclination is presented by an imbalanced information distribution. A method that has been used in previous works is Adaptive Synthetic Sampling (ADASYN) to enhance the classification accuracy by balancing the datasets, thus decreasing bias factors [25]. Table 1 shows that the original dataset is partially imbalance; therefore, we applied ADASYN to overcome this problem and to balance the dataset. After applying ADASYN sampling, the database then consisted of 354 microcytic, 327 normocytic, 312 macrocytic, 340 hypochromic, and 380 normochromic images of blood smears.

### 3.7. Features Reduction

**Figure 7:**
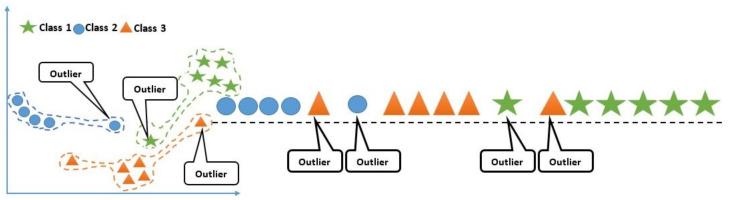
Feature reduction using Locality Sensitive Discriminant Analysis (LSDA).

To maintain variation among interclass data samples, it is necessary to reduce the dimensionality of an original dataset. We used Gray Level Cooccurrence Matrix (GLCM), Gray Level Run Length Matrix (GLRLM), and Gabor filter bank, which collectively produced 52 features for a single cell image in a blood smear image. Therefore, to reduce the dimensionality, a Locality Sensitive Discriminant Analysis (LSDA) [26] approach was applied separately to the features extracted from each cell (shown in Figure 7). We use LSDA because it is significant in the case where there are no sufficient training samples. LSDA uses local structures, and it is generally more important than global structure for discriminant analysis. LSDA determines a projection using the local manifold structure, which results in the maximization of the margin among data points from different classes at every local area. Various experiments on the existing datasets showed an improvement over the Linear Discriminant Analysis (LDA).

## 4. Classification

In different situations, varied instances show a tendency to a specific classification tool. Therefore, iterative experiments have been performed for the selection and determination of an optimal tool. The tools determined during the process were random forest and multilayer perceptual modal. These are ideal classification tools in this situation. For classification, the training data set was prepared with the extracted features mentioned above. The images were labelled with ground truth from the existing dataset images, containing Iron Deficiency Anaemia (IDA) images, mostly. The instances of dataset comprised of geometric morphological feature, GLCM, GLRL, and Gabor texture features are given in the features below. We used the classical machine learning methods as the overfitting and underfitting anomalies of pretrained deep learning algorithms suffer in accuracy of results due to less sufficient data in datasets. The performance of our classification technique is given in the Results Section 5.

## 5. Results

The results of the proposed work are as follows.

### 5.1. Dataset

The images we used in our work were collected from a local hospital. At the selected hospital, a high frequency of microcytic hypochromic patients were observed. The available datasets were also searched for such images, and a set of 150 images (comprising of about 80 hypochromic microcytes per image) were created based on ground truth, labeled by expert hematologists.

### 5.2. Qualitative Results

**Figure 8:**
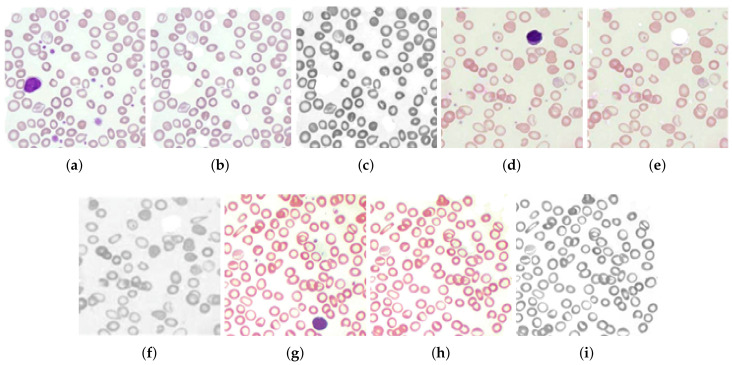
(**a**,**d**,**g**) Original RGB images of a sample blood smear, (**b**,**e**,**h**) removal of white blood cells (WBCs) from the blood smear images, and (**c**,**f**,**i**) enhanced gray-scale images of RBCs in blood smears.

**Figure 9:**
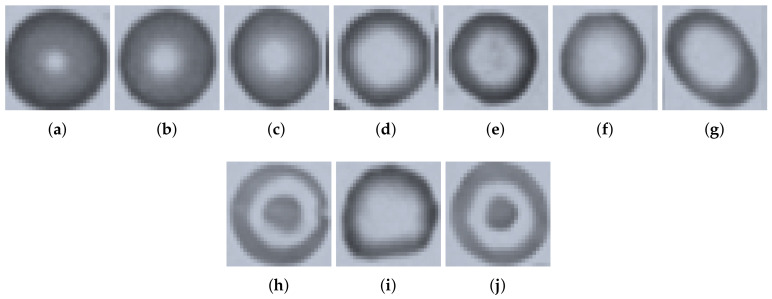
Classes of cells among RBCs (**a**,**b**) microcytic hypochromic cells, (**c**) microcytic narmochromic cells, (**d**) macrocytic hyperchromic cells (**e**,**f**) microcytic hyperchromic cells (**g**) macrocytic Hyperchromic cells, and (**h**,**j**) codocytes or taget cells.

**Figure 10:**
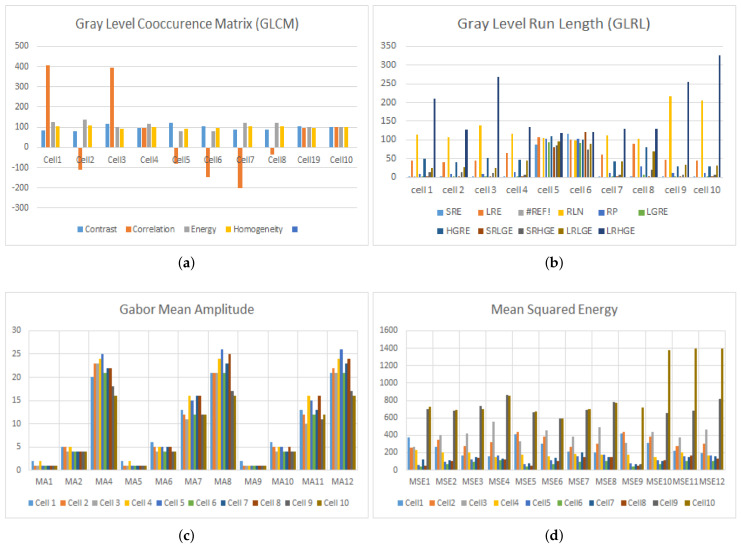
Graphs showing the results of four types of texture features, (**a**) GLRLM (**b**) GLCM (**c**) GMSE (**d**) GMA.

Hypochromic microcytes are the cells we are interested in for segmentation, the cell with a more pallor area and smaller than normal mean sizes of RBCs (Figure 8). These types of cells have less pixel area with more hole area proportionality. A hole containing cells was assumed to be a pallor cell and was less chromatic than the cell having no hole. The chromaticity factor is inversely proportional to the proportionality of central pallor: the bigger the central pallor, the less chromatic the cell will be and the cell will have a big hole in it after carrying out binarization operations, and hence, the concavity of the cell will also be more. The qualitative results of the GLCM, GLRL, Gabor mean amplitude, and Gabor mean square energy are given in Table 2, Table 3, Table 4 and Table 5. The qualitative results are also demonstrated in Figure 8 and Figure 9. The sample cells for which the features are extracted and demonstrated in Table 2, Table 3, Table 4 and Table 5 are shown in Figure 10.

### 5.3. Quantitative Results

This work has two main purposes: one is to segment the red blood cells accurately and then to detect the microcytic hypochromic cell. To evaluate the detection algorithm, the precision, recall, F1-score and accuracy metrices were calculated (using Equations (28)–(31)), where TP, TN, FP, FN are true positive, true negative, false positive, and false negative, respectively. To evaluate the detection algorithm, the precision, accuracy, recall, and F1-score matrices were calculated. The performance-wise results of different algorithms are given in Table 6.

(28) $$Precision=\frac{FP}{TP+FP}$$

(29) $$Recall=\frac{TP}{TP+FN}$$

(30) $$F1Measure=2\nabla \frac{precision\nabla Recall}{precision+Recall}$$

(31) $$Accuracy=\frac{TP+TN}{TP+TN+FP+FN}$$

## 6. Conclusions

The goal of this study is to develop and improve a robust algorithm for the analysis of blood smear images for the classification of microcytic hypochromic anaemia. Many studies are present in the literature that focus on the classification of blood cells, but there are very rare studies found on the classification of normal and abnormal blood slides as a specific anemic disease. Moreover, the existing state-of-the-art techniques are very expensive and their operation is very difficult. Our proposed system has a reasonable accuracy rate and processing time. The proposed system is capable of detecting various chromatic status of blood and accurately estimates the boundary pixels of RBCs at diverse photographic conditions. The results showed that our algorithm is a better automatic segmentation methods for blood smear images. The geometric and three potential texture features of RBCs, i.e., GLCM, GLRL, and Gabor texture features at 4 different degrees of scan (3, 6, 9, and 12) and 3 orientations θ (30${}^{\circ }$, 60${}^{\circ }$, and 90${}^{\circ }$) were extracted. After feature extraction and the feature reduction technique, the features were then put in two powerful machine learning algorithms (random forest and multilayer perceptron) using ensemble learning technique. The overall classification accuracy was 96%. Which was compared to the existing techniques and were found better for classification. The present work may be extended to a 3D volume estimation of blood cells, which is necessary in finding the accurate blood indices like Mean Corpuscular Volume (MCV), Mean Corpuscular Haemoglobin (MCH), and Mean Corpuscular Haemoglobin Concentration (MCHC). The proposed system may facilitate pathologists by getting quicker results with high True Positive (TP) and True Negative (TN) rates in the initial stage of diagnosis. It is user-friendly and easily operable with less expenses in terms of cost. Furthermore, the system may be made available on the web without any association of special equipment and user knowledge. Therefore, it can be considered an added value to the existing automated analysis blood smear tools.

## Figures and Tables

**Table 1:** Balanced datasets after applying Adaptive Synthetic Sampling (ADASYN).

Cell Type	Original	Synthetic	Total
Microcyte	157	197	354
Normocytes	270	57	327
Macrocytes	101	211	312
Hypochromic	157	150	340
Narmochromic	380	0	380

**Table 2:** Gray Level Cooccurrence Matrix (GLCM) features extracted from each cell in a sample image.

Cell No.	Contrast	Correlation	Energy	Homogeneity
Cell1	85.40	406.62	123.52	105.8
Cell2	80.02758	−112.132	136.3636	108.52
Cell3	116.9	394.59	100	93.68
Cell4	96.18	94.716	117.85	100.0
Cell5	121.1	−79.49	80	92.77
Cell6	106.2	−148.7	80	98.03
Cell7	86.6	−201.2	120	104.07
Cell8	88.12	88.12	120	104
Cell9	106.1	97.47	100	98
Cell10	100	100	100	100

**Table 3:** Gray Level Run Length (GLRL) features.

Cell No.	SRE	LRE	GLN	RLN	RP	LGRE	HGRE	SRLGE	SRHGE	LRLGE	LRHGE
Cell1	0.56	44.24	116.35	113.27	8.74	2.09	50.11	0.22	12.46	24.79	209.57
Cell2	0.7	40.25	126.78	108.01	8.69	2.26	39.37	0.29	14.12	25.91	127.59
Cell3	0.43	44.88	135.02	139.61	8.47	2.02	51.45	0.18	10.59	24.89	268.0
Cell4	0.5	65.18	137.38	115.35	12.93	2.62	48.2	0.23	7.83	45.05	134.07
Cell5	88.24	106.62	94.77	106.14	102.68	93.53	110.29	80.23	86.07	96.51	118.61
Cell6	115.4	100.85	94.51	98.77	103.26	92.73	99.82	120.77	73.02	88.5	120.18
Cell7	0.39	61.57	132.04	111.73	11.22	2.6	43.64	0.19	7.9	42.7	130.44
Cell8	1.25	89.76	103.12	103.36	29.92	7.01	81.33	0.59	19.3	70.39	130.52
Cell9	0.33	47.64	309.2	216.5	11.1	2.44	28.86	0.15	6.02	32.81	254.73
Cell10	0.37	45.41	355.65	206.29	10.46	2.56	28.17	0.18	5.99	31.69	325.68

**Table 4:** Gabor’s Mean Squared Energy (MSE).

Cell No.	MSE1	MSE2	MSE3	MSE4	MSE5	MSE6	MSE7	MSE8	MSE9	MSE10	MSE11	MSE12
Cell1	376	263	165.7	155.2	414.9	301.7	216.3	204.5	424.4	313.3	226.3	197
Cell2	258	350	274.6	316.8	436.3	380.9	264.9	306.7	439.7	382.9	273.3	299
Cell3	269	403	419.8	554.9	328.8	457.5	386.6	493.4	314.5	440.3	377.1	462
Cell4	234	205	207.9	153.3	177.8	157.8	186.5	174.7	173.5	153	200.3	172
Cell5	62	96	121.3	164.9	72.1	110.4	157.8	173.6	73.3	109.7	155.8	167
Cell6	45	70	91.5	113.5	40.8	70.5	93.3	104.4	41.5	72.4	102	105
Cell7	120	115	149.8	136	79.1	140	201.2	149.7	70.6	102.6	147.2	156
Cell8	50	101	138.6	120	51.8	101	149.7	146	54.2	113.4	163.6	131
Cell9	701	682	737.9	862.7	669	592	692	784	70	653.5	685.5	820
Cell10	730	695	700	850	670	590	701	770	715	1373.9	1396.1	1397

**Table 5:** Gabor’s Mean Amplitude (MA).

Cell No.	MA1	MA2	MA3	MA4	MA5	MA6	MA7	MA8	MA9	MA10	MA11	MA12
Cell1	2	5	11	20	2	6	13	21	2	6	13	21
Cell2	1	5	12	23	1	5	12	21	1	5	12	22
Cell3	1	4	11	23	1	4	11	21	1	4	10	21
Cell4	2	5	16	24	2	5	16	24	1	5	16	24
Cell5	1	4	13	25	1	5	15	26	1	5	15	26
Cell6	1	4	11	21	1	4	12	21	1	4	12	21
Cell7	1	4	13	22	1	5	16	23	1	4	13	23
Cell8	1	4	14	22	1	5	16	25	1	5	16	24
Cell9	1	4	12	18	1	4	12	17	1	4	11	17
Cell10	1	4	12	16	1	4	12	16	1	4	12	16

**Table 6:** Performance evaluation of each classification algorithm with statistical measurement tools.

Statistical	K-means Clustering (%)	Logistic Regression (%)	Naive Bayes (%)	Proposed Classifier (%)
Precision	81.1	86.4	87.1	92.3
Accuracy	80.7	86.2	88.3	93.2
Recall	80.3	83.2	84.3	95.4
F1-Score	79.8	82.1	84.1	94.1
